# Vessel diameters of 14 basal cerebral arteries assessed in 1000 digital subtraction angiographies

**DOI:** 10.1038/s41597-025-05908-7

**Published:** 2025-09-08

**Authors:** Till Gumbel, Cindy Richter, Christian Martin, Ulf Nestler

**Affiliations:** 1 Department of Neurosurgery, University Hospital Leipzig, Liebigstrasse 20, 04103 Leipzig, Germany; 2 Department of Neuroradiology, University Hospital Leipzig, Liebigstrasse 20, 04103 Leipzig, Germany; 3 ScaDS.AI, Faculty of Mathematics and Computer Science, Leipzig University, Humboldtstraße 25, 04105 Leipzig, Germany

**Keywords:** Brain, Epidemiology

## Abstract

Angiographic normative values for the size of intracranial vessels are difficult to obtain, since they vary with gender, height and weight. Cerebral angiography only is indicated in severe cerebrovascular diseases, which also can affect cerebral vessel diameters, impeding the definition of physiological values. To approximate “normal” values, over 1000 contemporary cerebral angiographies from a single neurovascular centre were analyzed. Diameters of 14 basal cerebral arteries, age at examination, gender and underlying disease were noted. The dataset (SPSS 29, IBM) comprises 1010 digital subtraction angiographies. For example, a significant difference (p < 0.001) in the size of the left carotid artery between male (3.23 mm, n = 361, sd = 0.49) and female (3.09 mm, n = 645, sd = 0.52) patients is found. The data can be used to compute intraindividual indices in given diseases, e.g. whether an enlarged diameter of the right media, calculated as ratio to the left media or to the ipsilateral carotid artery, is associated to cerebral aneurysms. The dataset allows for training of machine learning programs, e.g. to predict ischemic stroke or cerebral hemorrhage.

## Background & Summary

Hemorrhagic stroke occurs with a sudden onset and can result in severe neurologic deficits. Emergency treatment involves close collaboration of a number of medical specialists, such as neurologists, neuroradiologists or neurosurgeons, often followed by a prolonged stay on an intensive care unit. To detect the potential source of bleeding, imaging of cerebral vessels is performed, if feasible, by cerebral digital subtraction angiography (DSA).

Potential bleeding sources comprise cerebral aneurysms, arteriovenous malformations or cavernoma, the risk factors for bleeding can be elevated blood pressure or smoking habits. When asymptomatic vascular malformations are detected incidentally, individual patient counseling is needed, weighing the treatment risk against the bleeding risk during the natural course of the lesion^1^.

The data presented here allow division into a group of patients with hemorrhage versus a group without bleeding. A detectable difference in vessel diameters or vessel indices then could support patient counseling. For this purpose, the dataset will enable training of artificial intelligence in the context of machine learning, which is probably apt to provide deeper insights with more ease^2^.

On the other hand, following hemorrhagic stroke, vessel diameters can change considerably due to cerebral vasospasm. The downstream cerebral tissue is then at risk for ischemic deficits, requiring early prophylactic treatment on an intensive care unit. In these cases, the detailed knowledge of vascular diameters can help in treatment decisions^3^.

In the dataset provided here, cerebral angiographic images from a single centre were identified by searching the in-house, computer-based, inpatient and outpatient records from the neurosurgical clinic. Upon identification of a patient who had undergone cerebral angiography, the corresponding pictures were retrieved from the neuroradiologic department and transferred to an individual workstation for analysis.

## Methods

The retrospective, fully anonymised analysis of angiographic images for scientific purposes and publication had been approved by the local ethic-committee (#489/22-ek). A consent waiver has been provided.

Age and gender of the patient, as well as the disease leading to angiographic examination of cerebral vessels were collected from the computer-based patient records.

Vessel diameters of 14 basal cerebral arteries were obtained by one examiner (TG) using the in-house radiology viewer on an individual workstation. A well-contrasted arterial phase image was chosen and details were magnified by hand to a convenient size to position the measuring tool in a straight part of the vessel, near to the corresponding bifurcations. After parallel lining of the tool with the vessel walls, the diameter was read out in mm. The diameter calculation by the software of the viewer (IDS7 version 25.1, Sectra AB, Linköping, Sweden) is based on the image pixel size and the radiologic magnification factor, derived from the relation of the distances of the x-ray source to the patient and to the detector.

Except for the communicating posterior arteries, which were assessed in the lateral projection, all artery diameters were measured in the anterior-posterior (ap) projection (Figs. 1–8).

**Fig. 1:**
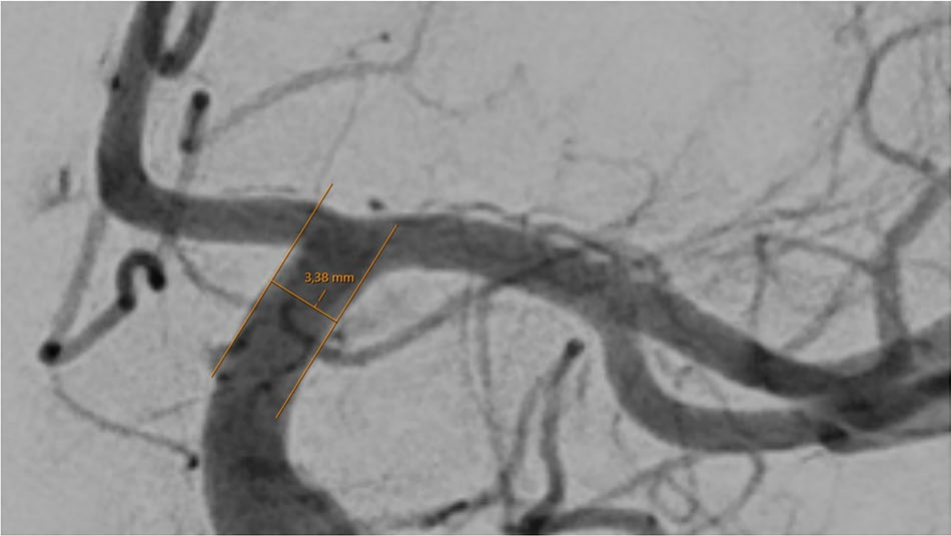
Assessment of the terminal segment of the left carotid artery. The caliper is placed 3 to 5 mm proximal to the carotid T and angled until parallel lining of its branches with the adjacent vessel walls is achieved. DSA, ap-projection, the diameter reads 3.38 mm.

**Fig. 2:**
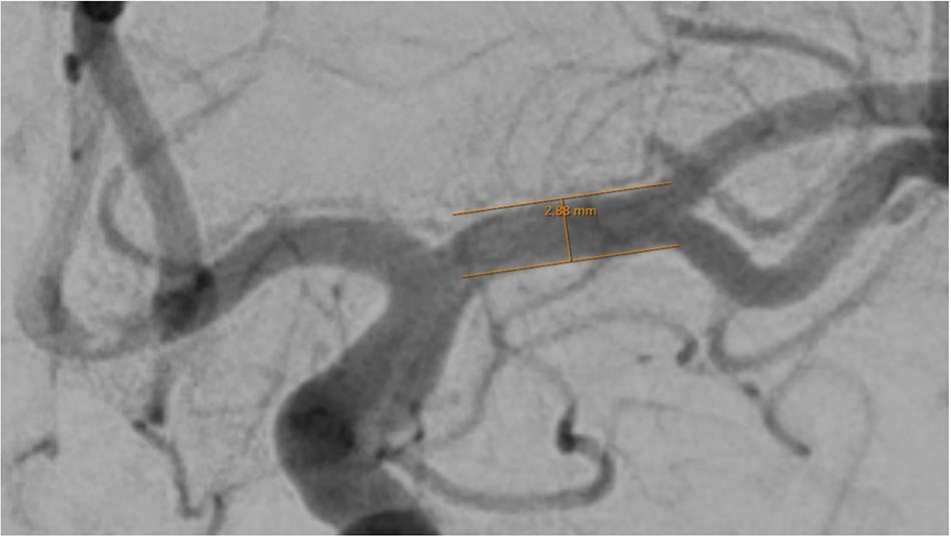
Assessment of the M1 segment of the left middle cerebral artery. The caliper is placed 3 to 5 mm distal to the carotid T and angled until parallel lining of its branches with the adjacent vessel walls is achieved. DSA, ap-projection, the diameter reads 2.88 mm.

**Fig. 3:**
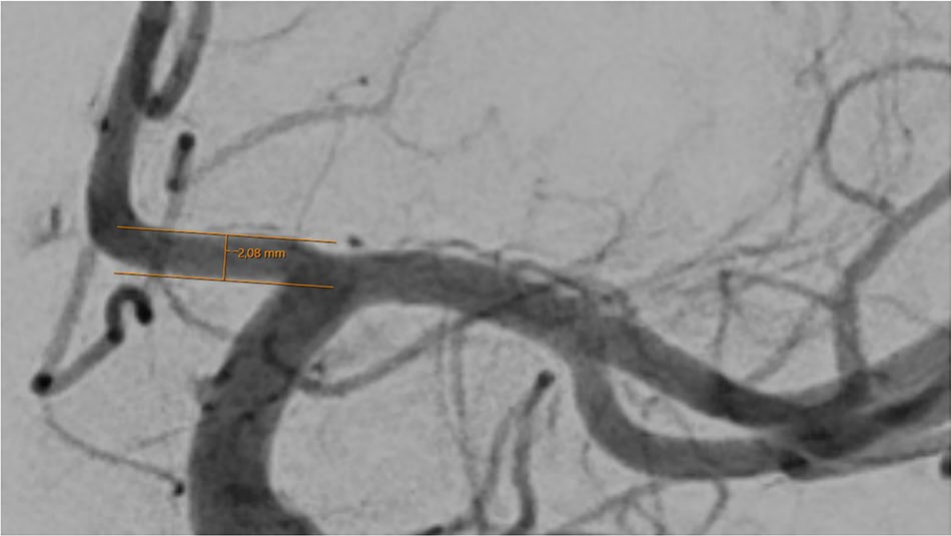
Assessment of the A1 segment of the left anterior cerebral artery. The caliper is placed 3 to 5 mm distal to the carotid T and angled until parallel lining of its branches with the adjacent vessel walls is achieved. DSA, ap-projection, the diameter reads 2.08 mm.

**Fig. 4:**
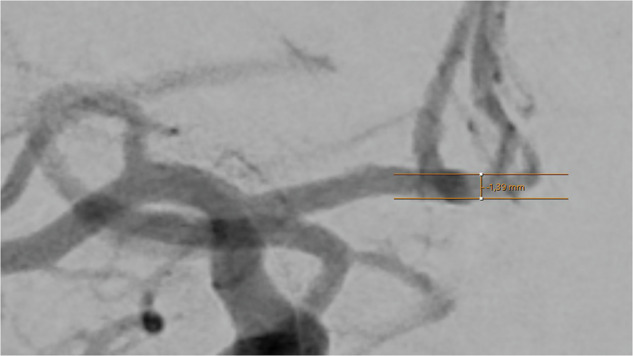
Assessment of the anterior communicating artery, injected from the right side. The caliper is placed medially to both A2 sections and angled until parallel lining of its branches with the adjacent vessel walls is achieved. DSA, ap-projection, the diameter reads 1.39 mm.

**Fig. 5:**
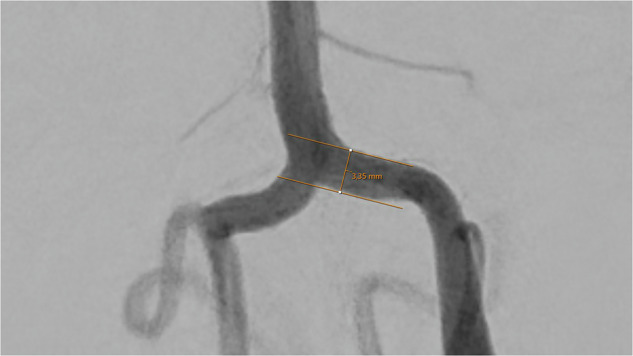
Assessment of the left vertebral artery, injected from the left side. The caliper is placed 2 to 3 mm proximal to the junction of the vertebral arteries forming the basilar artery, and angled until parallel lining of its branches with the adjacent vessel walls is achieved. DSA, ap-projection, the diameter reads 3.35 mm.

**Fig. 6:**
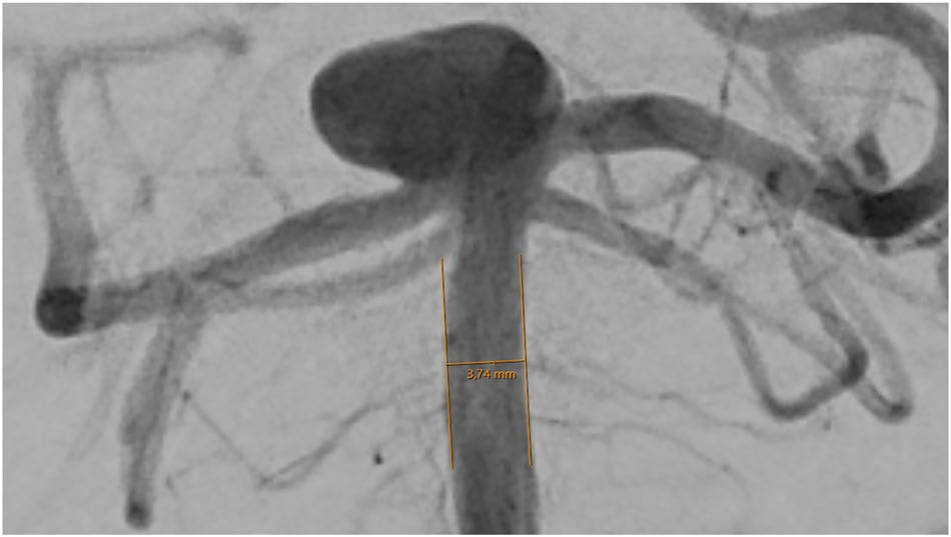
Assessment of the basilar artery. The caliper is placed on a straight part of the artery, in between the vertebral junction and the basilar tip, then angled until parallel lining with the adjacent vessel walls. DSA, ap-projection, the diameter reads 3.74 mm. A large aneurysm of the basilar tip can be seen bulging to the right side.

**Fig. 7:**
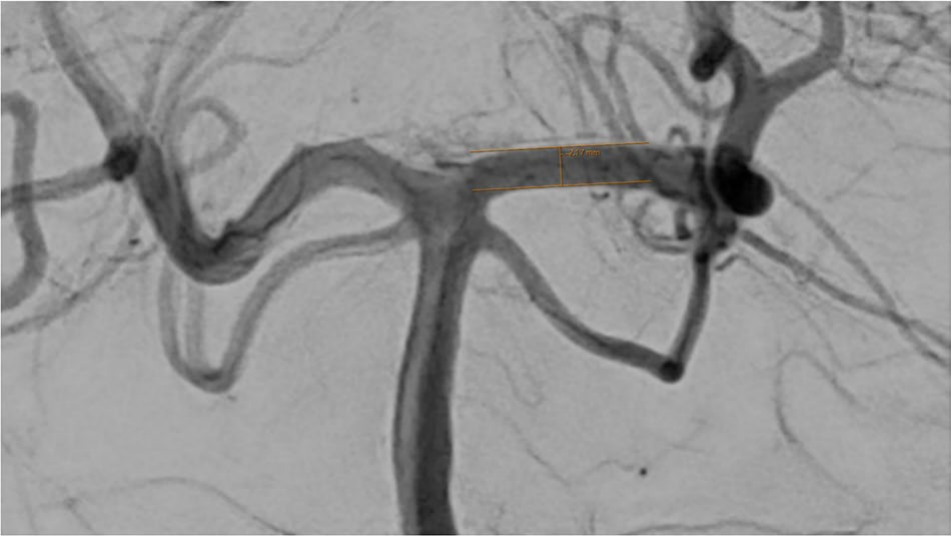
Assessment of the left posterior cerebral artery, injected from the left side. The caliper is placed 3 to 5 mm distal to the basilar tip and angled until parallel lining of its branches with the adjacent vessel walls is achieved. DSA, ap-projection, the diameter reads 2.17 mm. Washout flow on the right side of the basilar artery.

**Fig. 8:**
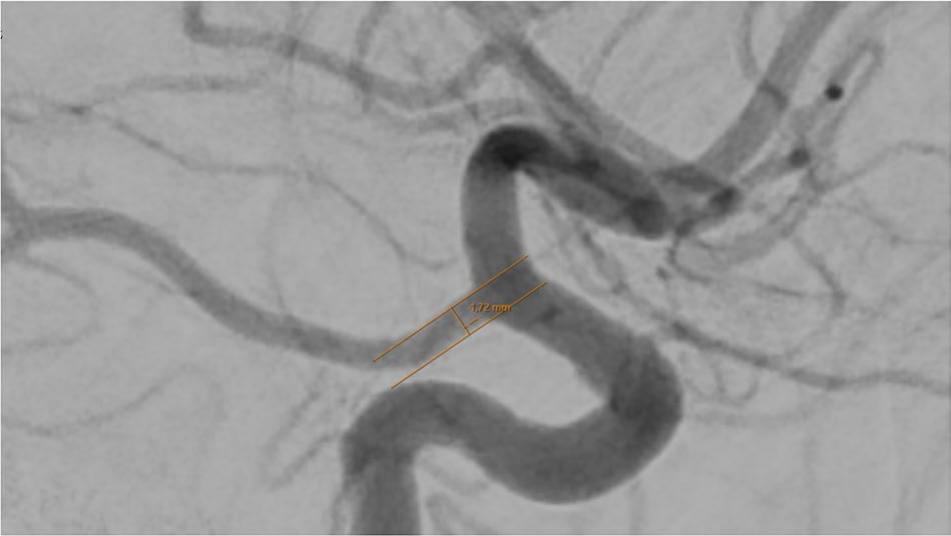
Assessment of the posterior communicating artery, injected via the ipsilateral carotid artery. The caliper is placed 2 to 3 mm distal to the origin of the vessel from the carotid artery, and then angled until parallel lining with the vessel walls. DSA, lateral projection, the diameter reads 1.72 mm.

Patient data and vessel diameters were noted as raw data in a SPSS datasheet without further processing (SPSS version 29, IBM Corp., Armonk NY, USA).

## Data Records

The SPSS datasheet is accessible in the repository Zenodo under the following link: https://zenodo.org/records/15222357^4^.

To anonymize the data, name and birthday of the patients were removed. The patient age is given in years, disclosing the age at the time of angiographic examination of the cerebral vessels. The gender (m/f) refers to the sex detected at birth (Table 1).

**Table 1:** Parameters, dimensions and categories.

parameter	dimension	value	category/description
Age	[years]		
gender		m	Male
		f	Female
DSAdays	[days]		number of days in relation to latest dataset
indication		1	aneurysmal subarachnoid hemorrhage
		2	incidental aneurysm
		3	subarachnoid hemorrhage without source
		4	arteriovenous malformation (without/with hemorrhage)
		5	arteriovenous fistula
		6	intracerebral hemorrhage
		7	cavernoma
		8	other
<…>_diameter	[mm]	>0	vessel diameter
		0	missing value “not injected”, “not filled”
		−1	missing value “not assessable”

The oldest angiography assessed, was performed in November 2003, 7183 days before the most recent, which was done in July 2023. The last examination date was set to day = 0. The column DSAdays gives the number of days in relation to the latest angiography, the negative value indicating how many days the angiography lies before this date.

The indications and diagnoses leading to cerebral digital subtraction angiography are classified into 8 main groups (Table 1).

The abbreviations used for the different vessel parts are shown in Table 2. The diameter of the vessels is given in mm. Missing values for the diameters are defined as “0” when the vessel had not been injected or did not fill during angiography, e.g. because of aplasia. Missing values “−1” disclose an unmeasurable vessel after injection, e.g. because of overlying structures such as an aneurysm.

**Table 2:** Abbreviation list of measured arteries.

left_ACI_diameter	left carotid artery
right_ACI_diameter	right carotid artery
left_Media_diameter	left middle cerebral artery
right_Media_diameter	right middle cerebral artery
left_Anterior_diameter	left anterior cerebral artery
right_Anterior_diameter	right anterior cerebral artery
Anterior_communicating_diameter	anterior communicating artery
left_Vertebral_diameter	left vertebral artery
right_Vertebral_diameter	right vertebral artery
Basilar_diameter	basilar artery
left_Posterior_diameter	left posterior artery
right_Posterior_diameter	right posterior artery
left_P_comm_diameter	left posterior communicating artery
right_P_comm_diameter	right posterior communicating artery

## Technical Validation

To rule out interobserver bias, all measurements were performed by the same examiner, as part of a data collection for a medical doctoral thesis on cerebral aneurysms (TG). After being introduced into the methodology by a senior neurosurgeon (UN), assessment of inter-rater variability by comparison of the results of the two examiners, assessment of intra-rater variability by re-measuring the first datasets, and final comparison of the 100 oldest to the 100 most recent angiographies did not result in significant differences between the mean vessel sizes.

Vessel diameter calculation was standardized by the software of the viewer (IDS7 version 25.1, Sectra AB, Linköping, Sweden). Taken into account were the image pixel size and the radiologic magnification factor, including the relation of the distances of the x-ray source to the patient and to the detector.

Altogether, 1010 angiographies from 1010 patients with a mean age of 53.36 years (sd 14.3, range 0 to 84 years) were analyzed. The dataset included 648 female (53.9 years) and 362 male patients (52.4 years). Most patients had to undergo angiographic examination in conjunction with a cerebral aneurysm, either for an incidental aneurysm (42.4%) or following aneurysmal subarachnoid hemorrhage (34.4%, Table 3).

**Table 3:** Indications for DSA (n = 1010).

	Indication	number	frequency %
1	aneurysmal subarachnoid hemorrhage	347	34.4
2	incidental aneurysm	428	42.4
3	subarachnoid hemorrhage without source	43	4.3
4	arteriovenous malformation (without/with hemorrhage)	117	11.6
5	arteriovenous fistula	6	0.6
6	intracerebral hemorrhage	17	1.7
7	Cavernoma	12	1.2
8	Other	40	4.0

To assess a potential influence of evolving angiographic techniques, vessel diameters were related to the date of angiography by linear regression for each of the 14 vessels (Table 4). In sum, regarding the r^2^ values and the calculated regression coefficients close to zero, no technical bias such as systematic larger vessel measurements in older or in more recent angiographic examinations was found.

**Table 4:** Number of assessable vessel diameters, and results of linear regression analysis in function of the date of angiography.

vessel	assessable (n)	mean diameter [mm]	standard deviation	regression coefficient (x 10^−5^)	r^2^ value
left ACI	1006	3.1418	0.517	4.468	0.022
right ACI	1007	3.1592	0.568	6.060	0.033
left Media	1008	2.4502	0.398	4.758	0.042
right Media	1006	2.4639	0.408	4.308	0.032
left Anterior	1007	1.9399	0.423	4.175	0.028
right Anterior	989	1.8866	0.444	3.944	0.023
Anterior comm	466	1.5293	0.479	4.762	0.029
left Vertebral	933	2.6712	0.631	3.760	0.010
right Vertebral	655	2.4587	0.597	6.324	0.030
Basilar	1008	2.6257	0.567	2.712	0.007
left Posterior	1006	1.8080	0.537	0.661	0.0004
right Posterior	1001	1.7411	0.589	−0.604	0.0003
left Post comm	1002	1.1000	0.632	−0.487	0.0002
right Post comm	999	1.1605	0.661	0.159	0.00002

In the second column of Table 4 the number of measurable angiographic diameters for each vessel are given, reflecting how many arteries did not fill after the injection or were hidden by overlying structures.

## Data Availability

The SPSS datasheet is accessible without restrictions in the repository Zenodo under the following link: https://zenodo.org/records/15222357^4^.

## Code availability

For this study, no previously unreported custom computer code has been used to generate or process the data. The datasheet software was SPSS version 29 (IBM Corp., Armonk NY, USA).
